# Supplementary material for: Susceptibility of swine cells and domestic pigs to SARS-CoV-2
Source: Emerg Microbes Infect. 2020 Oct 20;9(1):2278–88. doi: 10.1080/22221751.2020.1831405 (PMC7594707; doi:10.1080/22221751.2020.1831405)
Supplement: Meekins_et_al_Supplemental_Materials-clean.docx [file TEMI_A_1831405_SM8625.docx]

Supplemental Table 1. Daily rectal temperatures (˚C) of pigs enrolled in the study.

|  |  | DPC | | | | | | | | | | | | | | | | | | | | | |
| --- | --- | --- | --- | --- | --- | --- | --- | --- | --- | --- | --- | --- | --- | --- | --- | --- | --- | --- | --- | --- | --- | --- | --- |
| Group | Pig # | 0 | 1 | 2 | 3 | 4 | 5 | 6 | 7 | 8 | 9 | 10 | 11 | 12 | 13 | 14 | 15 | 16 | 17 | 18 | 19 | 20 | 21 |
| Primary  Inoculated | 893 | 40.6 | 39.5 | 39.6 | 39.5 | 39.5 | 39.3 | 39.2 | 39.5 | 39.2 | 39.4 | 39.3 | 39.2 | 39.6 | 40.1 | 39.3 | 40.1 | 39.6 | 39.9 | 39.0 | 39.6 | 39.5 | 39.3 |
|  | 193 | 40.4 | 40.0 | 39.9 | 40.0 | 39.7 | 39.4 | 39.4 | 39.6 | 39.2 | 39.8 | 40.2 | 39.6 | 40.3 | 39.9 | 39.7 | 41.3 | 40.8 | 39.7 | 39.5 | 39.7 | 39.7 | 39.6 |
|  | 219 | 40.4 | 39.8 | 39.8 | 39.8 | 39.7 | 39.7 | 39.6 | 39.6 | 39.2 | 39.5 | 40.2 | 39.5 | 39.9 | 39.7 | 39.7 | 39.9 | 39.9 | 39.9 | 40.2 | 40.8 | 40.5 | 40.4 |
|  | 803 | 40.3 | 40.2 | 40.4 | 39.9 | 39.9 | 40.1 | 39.7 | 40.2 | 40.0 |  |  |  |  |  |  |  |  |  |  |  |  |  |
|  | 211 | 40.0 | 39.6 | 39.4 | 39.5 | 39.5 | 39.6 | 39.6 | 39.7 | 40.4 |  |  |  |  |  |  |  |  |  |  |  |  |  |
|  | 841 | 40.0 | 39.6 | 39.7 | 39.4 | 39.3 | 39.4 | 39.4 | 39.8 | 39.7 |  |  |  |  |  |  |  |  |  |  |  |  |  |
|  | 807 | 40.1 | 40.6 | 39.7 | 39.5 | 40.4 |  |  |  |  |  |  |  |  |  |  |  |  |  |  |  |  |  |
|  | 161 | 40.2 | 39.9 | 39.5 | 39.9 | 39.8 |  |  |  |  |  |  |  |  |  |  |  |  |  |  |  |  |  |
|  | 168 | 39.6 | 39.5 | 39.3 | 39.4 | 39.5 |  |  |  |  |  |  |  |  |  |  |  |  |  |  |  |  |  |
| Contact  Controls | 194 |  | 39.9 | 39.8 | 39.7 | 39.6 | 39.6 | 39.6 | 39.5 | 39.8 | 40.1 | 39.9 | 39.8 | 39.7 | 39.7 | 39.8 | 40.3 | 39.6 | 40.2 | 40.2 | 39.7 | 39.5 | 39.9 |
|  | 222 |  | 40.1 | 40.0 | 39.4 | 39.7 | 39.8 | 39.6 | 40.0 | 39.6 | 39.7 | 39.5 | 39.6 | 39.5 | 39.6 | 39.4 | 39.4 | 39.4 | 39.1 | 39.6 | 39.2 | 39.4 | 38.8 |
|  | 848 |  | 41.2 | 39.7 | 40.4 | 39.3 | 39.7 | 39.6 | 40.1 | 39.1 | 39.2 | 40.1 | 40.1 | 39.9 | 39.6 | 39.6 | 40.1 | 40.1 | 39.8 | 39.7 | 39.6 | 39.9 | 39.6 |
|  | 851 |  | 40.1 | 39.9 | 39.4 | 39.2 | 38.7 | 39.5 | 39.7 | 39.5 | 39.5 | 39.2 | 39.2 | 39.4 | 39.3 | 39.4 | 39.6 | 39.6 | 39.6 | 39.2 | 39.4 | 39.6 | 39.4 |
|  | 172 |  | 40.8 | 40.4 | 39.6 | 39.1 | 39.3 | 39.7 | 39.7 | 39.7 | 39.9 | 39.4 | 39.9 | 39.9 | 40.0 | 39.6 | 39.9 | 39.6 | 39.7 | 39.8 | 39.3 | 39.6 | 39.7 |
|  | 894 |  | 40.6 | 41.1 | 39.7 | 39.4 | 38.8 | 39.4 | 39.1 | 39.2 | 38.8 | 39.3 | 39.3 | 39.4 | 39.2 | 39.6 | 39.7 | 39.5 | 39.1 | 39.2 | 39.3 | 39.3 | 39.4 |
| Uninoculated  Controls | 201 |  | 40.1 | 40.0 | 40.2 |  |  |  |  |  |  |  |  |  |  |  |  |  |  |  |  |  |  |
|  | 183 |  | 40.1 | 39.6 | 39.7 |  |  |  |  |  |  |  |  |  |  |  |  |  |  |  |  |  |  |
|  | 238 |  | 40.1 | 39.6 | 39.2 |  |  |  |  |  |  |  |  |  |  |  |  |  |  |  |  |  |  |
